# Comparison of Twin Screw Derotation Type Versus Single Helical Blade Type Cephalomedullary Nail in Trochanteric Fractures in Geriatric Population

**DOI:** 10.7759/cureus.31557

**Published:** 2022-11-15

**Authors:** Sanjay Yadav, Raghul Dakshinamoorthy

**Affiliations:** 1 Orthopedics, Institute of Medical Sciences (IMS) Banaras Hindu University (BHU), Varanasi, IND

**Keywords:** geriatric, pfna2, nail, intertrochanteric, fracture

## Abstract

Introduction

The aim of this study was to compare the usage of two devices, the twin screw derotation type and the helical blade type, in intertrochanteric fractures in the geriatric population at a tertiary-level center.

Methods

Forty-six eligible patients with intertrochanteric fractures operated with the standard proximal femoral nail (PFN) or proximal femoral nail anti-rotation (PFNA2) were included for analysis. This was a retrospective analysis of prospectively collected data over the study period. Demographics, various operative parameters, outcome parameters, and complications were assessed. Medical management of osteoporosis was provided to all patients. The student t-test and chi-square test were used with SPSS V22.0 (IBM Corp., Armonk, NY) for statistical analysis.

Results

The mean follow-up was 14 months. The hospital stay and Harris hip score were similar in both groups, but the radiation exposure, surgical time, and blood loss were significantly less in the case of PFNA2. Screw cut-out was also not observed in our study.

Conclusion

Both PFNA2 and PFN are effective in treating unstable trochanteric fractures in terms of functional outcomes. However, PFNA2 is better because it requires less radiation exposure (p<0.05) due to single guidewire use, a short learning curve, less blood loss, shorter surgical time(p<0.05), and fewer complications. We suggest it to be the preferred implant in trochanteric fractures in the geriatric population and other age groups.

## Introduction

Proximal femoral fractures are extremely common in the geriatric population. In recent years, there is a significant increase in the incidence of intertrochanteric fractures due to the increase in life expectancy [1,2]. Surgical fixation is an optimal strategy for such fractures for early mobilization and recovery and to prevent problems associated with prolonged recumbence. The choice of implant in such fractures remains controversial for osteoporotic bone.

Many studies have been conducted comparing intramedullary versus extramedullary implants [3,4]. The intramedullary implant is reported to be better due to its better biomechanical properties, mini incisions, and less blood loss, although screw cut-out, the Z-effect, varus collapse, and more radiation exposure continue to be major concerns, as reported in the literature [5,6].

Both the proximal femoral nail (PFN) and the proximal femoral nail anti-rotation (PFNA2) are intramedullary devices with a 6-degree medial-lateral angle. PFN has two proximal screws, an anti-rotation screw, and a lag screw. PFNA2 has a single helical screw designed to achieve extra anchorage by the preservation of bone stock and compaction of cancellous bone around it during insertion. The helical blade is believed to provide rotational stability and compression as well.

Traditionally, PFN was meant for unstable intertrochanteric fractures, and PFNA2 was subsequently developed for use in osteoporotic fractures. We intended to compare them in similar patients, as PFN needs more accurate placement of two screws, which is sometimes difficult. Furthermore, the time factor and radiation exposure are significantly reduced with a single helical blade device. This is more surgeon- and patient-friendly. With these thoughts, we attempted to study the clinical and radiological outcomes of PFN versus PFNA2 fixation in osteoporotic intertrochanteric fractures in the elderly.

## Materials and methods

This retrospective study included all patients with unstable intertrochanteric fractures operated with PFN or PFNA2 from May 2016 to May 2018 in the orthopedic department of our institution. Patients with isolated unilateral intertrochanteric fractures of the AO 31.A2 and AO 31.A3 types with a Singh index >3 were included for analysis. Patients with stable fractures, pathological fractures, multiple fractures, and severe comorbidities, such as cardio-respiratory problems, were excluded.

A total of 66 patients operated on with PFN or PFNA2 were considered. Twenty patients were excluded due to associated injuries such as head injuries and chest or abdominal trauma. The remaining 46 patients were evaluated. Out of 46 patients, 23 were operated on with PFN (Nebula, S.H Pitkar OrthoTools, Maharashtra, India), and the remaining 23 patients with PFNA2 (AO type, Medtronic, Dublin, Ireland). The cases with PFNA2 numbered 23, and an equal number of cases were included from the PFN cases.

The implant options were explained to the patient in all cases, and the implant chosen by the patient was used per affordability. Age was not the sole consideration for the implant choice. However, for severe radiological osteoporosis, PFNA2 was suggested and used accordingly. Demographics such as age, gender, and comorbidities were recorded. Each fracture was classified per the AO classification system.

All patients were operated on a fracture table under spinal anesthesia. Preoperative antibiotics were given to both groups 30 minutes before the incision. During the operations, surgical time, the number of image intensifier shots used, and blood loss were recorded. Isometric quadriceps and ankle pumps were started as soon as possible. All patients were mobilized, such as sitting and turning in bed, from the second postop day and allowed toe-touch weight bearing with the help of a walker as tolerated. Further weight bearing was allowed per union progression. The rehabilitation protocol was similar in both groups. No postoperative thromboprophylaxis was used.

Clinical and radiological assessment was done at the six-month and one-year follow-ups. Functional assessment was conducted with the Harris hip score at the one-year follow-up. Medical management of osteoporosis was provided to both groups. In the PFN group, bisphosphonates were used primarily, and in the PFNA2 group, teriparatide injections were given to most patients, with calcium and vitamin-D supplementation. This was based on the option chosen by the patient. Statistical analysis was done using SPSS V22.0 software (IBM Corp., Armonk, NY). Informed consent was obtained from all study subjects.

## Results

A total of 46 patients were evaluated, with 23 in each group, PFN and PFNA2. The mean ages were 68.4±3.5 years (PFN group) and 70±4.3 years (PFNA2 group). There were nine males and 14 females in the PFN group and 10 males and 13 females in the PFNA2 group. All fractures were considered unstable per the AO classification. We could achieve a reduction in all cases using the closed method or the joystick method at the screw insertion site. Road traffic accidents and home falls were the major causes of injury (88%). In the cases of falls at home or trivial trauma, osteoporosis was considered a contributory factor.

Various operative and follow-up parameters, including surgical time, blood loss, number of fluoroscopic images taken, and complications noted, are charted in Table 1.

**Table 1:** Comparison of various parameters in two study groups (PFN and PFNA2) PFN: proximal femoral nail; PFNA2: proximal femoral nail anti-rotation

Parameter	Group 1: PFN (n=23)	Group 2: PFNA2 (n=23)	P value
Demographics			
Age (yrs)	68.4±3.5	70.0±4.3	-
Gender (females)	14/23	13/23	-
Operative parameters			
Surgery time (min)	37.0±5.2	28.6±3.9	<0.01
Blood loss (ml)	93.7 ± 18.3	73.2 ± 14.5	<0.01
Number of IITV shots	34.6 ± 5.2	20.9 ± 4.9	<0.01
Post-op electrolyte imbalances (Hyponatremia)	3/23	1/23	-
Complications			
Screw cut-out	2	-	-
Z effect	1	-	-
Lateral protrusion of implant	-	1/23	-
Screw tip positioning (TAD ≥25mm)	3/23	2/23	-
Centro-central and centro-inferior positioning	20/23	22/23	-
Follow up parameters			
Limb shortening >1cm	2/23	-	-
Varus malalignment	2/23	1/23	-
Persistent pain	6/23	2/23	-
Pre-injury activity return	16/23	14/23	-
Support for walking (at 1yr)	20/23	18/23	-
Harris hip score	82.1±7.7	85± 5.8	0.15
Full weight bearing at 6 mo	12/23	17/23	<0.05

The tip-apex distance was centro-central or centro-inferior in all but one PFNA2 case in which it was slightly superior. The screws had more variability in the PFN group, with less-than-optimal positioning in four cases (18%).

There was no incidence of infection in either group. The mean hospital stay was similar for both groups (3.5 days). There were two cases of the Z-effect and one case of nail breakage in the PFN group. One instance of lateral protrusion of the helical blade was seen in the PFNA2 group.

**Figure 1:**
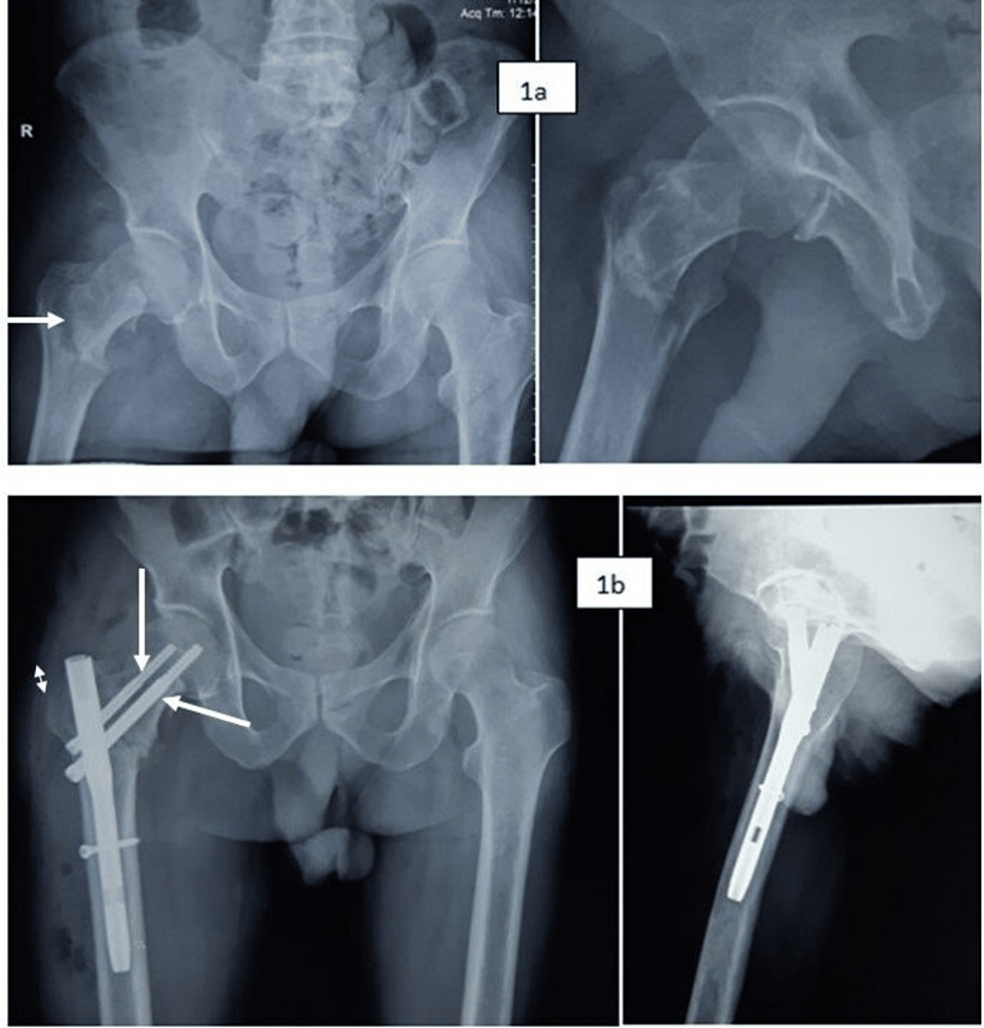
a,b. Case example showing fixation with PFN with a lag screw and a derotation screw Obtaining the optimum position of both screws remains a challenge. Also, the proximal end of the nail remains prominent above the greater trochanter in many cases owing to anatomical differences. PFN: proximal femoral nail

**Figure 2:**
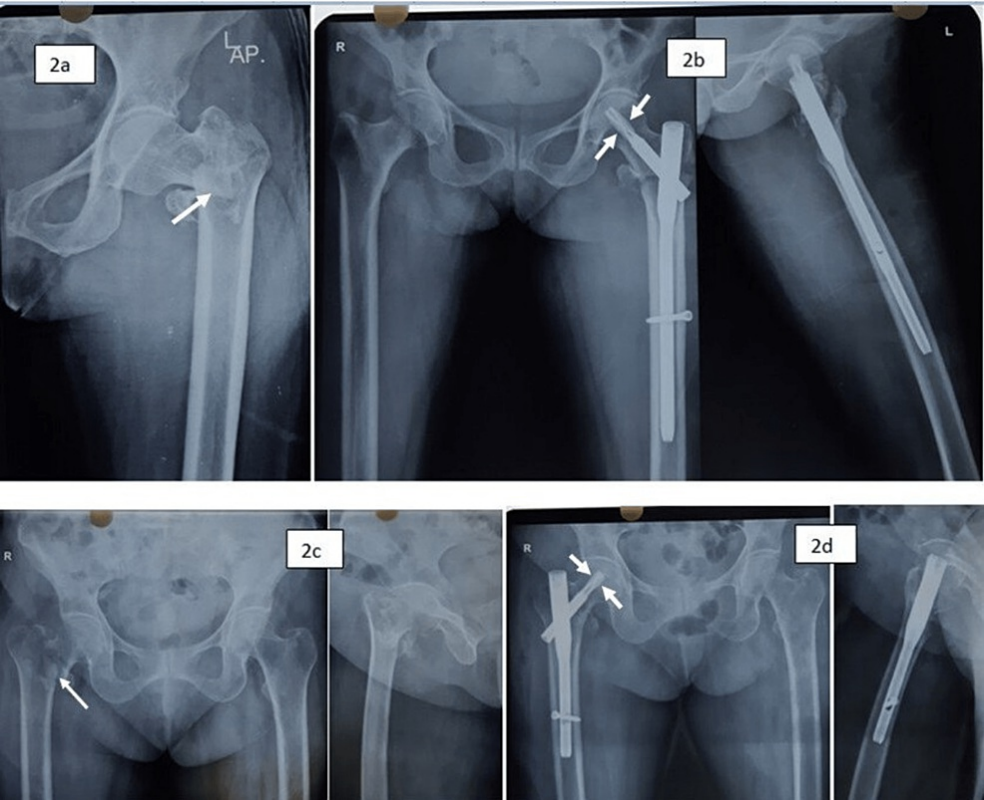
a. Case example showing fixation with PFNA2 (standard length - 240 mm). In this comminuted pertrochanteric fracture, the standard length was used so that distal locking can be done with the aiming device; b. Fixation with PFNA2 (short length - 170 mm) In this case, the helical blade in the neck seems to be in the center but it is slightly superior in the head. We aim to keep the upper edge of the helical blade in the neck center, which gives a slightly centro-inferior blade position. PFNA2: proximal femoral nail anti-rotation

The mean Harris hip score at one year was comparable between the groups. Only 60% of the cases regained near-normal preinjury status. At the one-year follow-up, 90% were still using support. Case examples are shown in Figures 1, 2.

## Discussion

Intertrochanteric fractures are very common in the elderly age group, and if not treated early, they may lead to various comorbidities associated with prolonged bed rest. Early fixation and mobilization are needed to prevent such comorbidities. Strong early stabilization can be considered the first step in the rehabilitation of such patients. Intertrochanteric fractures in the setting of osteoporotic bone remain a challenge for orthopedic surgeons [7].

Intramedullary implants have proven to be biomechanically better when compared with extramedullary devices. However, enhancement efforts are ongoing, like cement augmentation and improvements in implant designs. PFNA2 was designed considering these points. Its helical blade compacts the cancellous bone, as it is hammered and not drilled or reamed. Moreover, it is said to provide a large contact area between the blade and the bone, leading to better hold and stress distribution [8].

The average ages in our study were 68.4 years (PFN) and 70 years (PFNA2), which were comparable with other studies [9,10]. As in other studies, females were more commonly involved (58.7%), with equal distribution in the two study groups. Road accidents and falls at home had no significant differences in fracture pattern per AO classification, even though fractures due to road accidents tend to be of the AO 31.A3 type.

The difference in surgical time was 18 minutes on average between the groups in our study, which was statistically significant. A few investigators have reported the operative time as 40 minutes less in PFNA2 groups, but they also considered anesthesia time [9]. Others compared PFNA2 with the dynamic hip screw and reported less operative time and fewer complications with the use of PFNA2 in a cohort study [11,12]. Blood loss was significantly less in the PFNA2 group in the present study. The findings of reduced surgical time and blood loss were also corroborated by different studies [10]. Reduced surgical time, less blood loss, and reduced anesthesia medications may combine to lead to better recovery and rehabilitation.

Two instances of screw back-off (Z-effect) and one implant breakage were noted in the PFN group. One lateral protrusion of the sliding blade was noted in the PFNA2 group. There was no cut-out in the PFNA2 group. Fewer cut-out rates with the use of PFNA2 in the elderly compared with PFN have also been described in other studies [13]. However, lateral protrusion of the helical blade was reported in PFNA2, and 83% of cases regained preoperative mobility in one study compared with 60% in the present study [14].

The findings in terms of reduced surgical time, hence, reduced blood loss and radiation exposure, as determined by the number of fluoroscopic shots, were statistically significant in the present study. This was because the helical blade needed a single guide wire to be inserted, resulting in more space in the neck than two guidewires in the case of PFN with a smaller femur size due to Asian ethnicity. The attachments were also radiolucent, facilitating the procedure in the case of the standard imported PFNA2 implant in our study. Only the lateral cortex needed to be reamed in most cases. The tapping guide had markings on it, which also reduced the image intensifier usage. However, other studies could not find any differences in implant positioning between PFN and PFNA2 [15].

No postoperative infection, thromboembolism, or any other complications were noted. The mean Harris hip score was comparable in both study groups. Around 90% of the patients were using some kind of support in the form of a walker or a stick at the end of the one-year follow-up. This was more of a precautionary behavior to avoid falls and re-injury.

Considering the limitations, this was a single-center comparative study with small sample size and short-term follow-up. The implant choice was dependent on the patient’s ability to pay or insurance status. The PFN implant was Indian-made, and the PFNA2 device was imported, which could have affected the surgical time due to the variability in ease of instrumentation. The use of the Singh index instead of dual-energy X-ray absorptiometry to assess osteoporosis due to financial and practical constraints was another limitation, but this did not change the management. In future studies, the use of PFNA2 can be considered even in young subjects due to its time and blood-preserving advantages other than that in osteoporosis.

## Conclusions

Both PFNA2 and PFN were effective in treating unstable trochanteric fractures with equivalent functional outcomes. However, PFNA2 seemed better, as it required less radiation exposure (p <0.001), had a shorter learning curve, less blood loss, shorter surgical time (p<0.001), and fewer complications. This also led to faster recovery and rehabilitation. For these reasons, it is becoming the preferred device in intertrochanteric fractures in old age and may also be considered in younger patients for similar reasons.
